# Unusual esophageal foreign body in neonates: A case report

**DOI:** 10.1016/j.ijscr.2021.106236

**Published:** 2021-07-27

**Authors:** Dian Adi Syahputra, Dikki Drajat Kusmayadi, Vita Indriasari, Fransiska Kusumowidagdo

**Affiliations:** a Pediatric Surgery Division, Department of Surgery, Faculty of Medicine, Syiah Kuala University/Dr. Zainoel Abidin Hospital, Banda Aceh, Aceh, Indonesia; b Pediatric Urogenital Fellowship, Pediatric Surgery Division, Faculty of Medicine, Padjadjaran University/Hasan Sadikin Hospital, West Java, Indonesia; c Pediatric Surgery Digestive Consultant, Pediatric Surgery Division, Faculty of Medicine, Padjadjaran University/Hasan Sadikin Hospital, West Java, Indonesia; d Pediatric Surgery Urogenital Consultant, Pediatric Surgery Division, Faculty of Medicine, Padjadjaran University/Hasan Sadikin Hospital, West Java, Indonesia; e Pediatric Surgery Department, Faculty of Medicine, Airlangga University, Soetomo Hospital, East Java, Indonesia

**Keywords:** Esophageal foreign body, Neonates, Diagnosis, Flexible endoscopy, Prevention

## Abstract

**Introduction and importance:**

Esophageal foreign body mostly occurs in children aged 6 months to 5 years old. In neonates (babies less than 28 days old), such report is extremely rare. In this case, we report the first esophageal foreign body in neonates without any symptoms.

**Case presentation:**

A 28-day-old baby boy, with normal body weight, from a low socio-economic status family, came to us with a history of ingested foreign body. No sign of unconsciousness, excessive saliva, dysphagia, and respiratory distress. The chest X-ray revealed a radiopaque foreign body in the upper third of the esophagus. The patient underwent extraction of the foreign body using a flexible endoscope under general anesthesia. We found a 1.9 cm in diameter pendant with no sign of bleeding nor inflammation in the esophageal lumen. After the foreign body removal, the patient is in good condition and recovered uneventfully.

**Clinical discussion:**

Foreign body in children under 6 months old is very rare. A high index of suspicion for foreign body ingestion must be considered in unattended children from lower socio-economic status, primarily if witness statements are present and confirmed with radiological examination. Most common impaction site is at the level of the cricopharyngeus muscle. Currently, flexible endoscopy is the standard for foreign body removal in children.

**Conclusion:**

High index suspiciousness, witness statements and radiological examination are the important points in diagnosing ingested foreign body in neonates. Clinicians are required to provide education to parents to supervise their children when playing together.

## Highlights

• Esophageal foreign body extremely rare in neonates
• Most common without symptoms due to the diet consists of full fluids
• Witness statements and radiological examination are an important point in diagnosis.
• The impaction of foreign body is strong indication for foreign body removal.
• Flexible endoscopy is the standard for foreign body removal in children.

## 1 Introduction

The curiosity of a baby/child makes them brought to the emergency department. One of the common cases is that the ingested foreign bodies often occur in children aged 6 months to 5 years which can cause morbidity and mortality [1]. In a 2018 report from the American Association of Poison Control Centers' National Poison Data System, there were 66,519 cases of ingestion of foreign bodies in children under 5 years of age [2], [3]. It is more frequent in boys than girls; it varies according to socio-cultural characteristics. In general, objects that commonly swallowed are metal objects (e.g. coins, paper clips, batteries, needles), and non-metal objects (e.g. food, wooden, plastic pieces of toys). Early intervention is maybe required [1].

Ingestion of foreign bodies in the esophagus has various symptoms such as vomiting, dysphagia, increase in saliva production, changes in the daily diet habit resulting from loss of appetite, or hematemesis [1]. In some institutions, the management of ingested foreign bodies is a shared specialty between pediatric surgery, pediatric gastroenterology, and pediatric otolaryngology. Ingestion of foreign bodies in children under 6 months is very rare [2].

In this case report, we report a 28-day-old baby boy ingested a foreign body. This case report is presented in line with SCARE's 2020 Criteria [4].

## 2 Case presentation

A 28-day-old baby boy, weighing 4.2 kg, from low socio-economic status, was brought to the Primary Health Care (PHC) because he had swallowed his mother's pendant about 16 h ago. This happened when his older brother was playing and took his mother's pendant, and accidentally gave it to the patient. The patient was given breast milk twice after swallowing the pendant without any symptoms. At the time in the PHC, the patient was compos mentis, crying loudly with 99% SpO_2_.

**Fig. 1:**
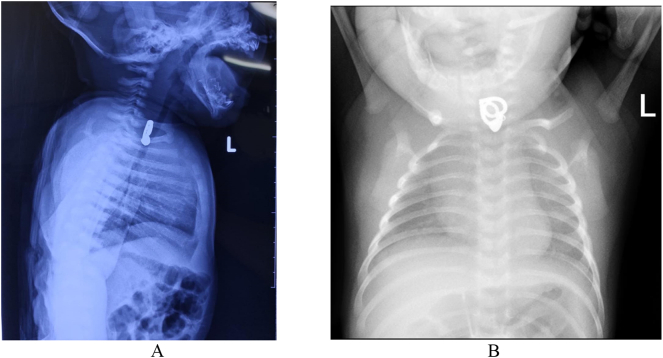
A and B. Chest X-ray show the foreign body at the level of the 7th Cervical vertebrae - 1st Thoracal behind the trachea.

The General Practitioner then performed an Antero-Posterior and lateral chest x-ray examination. There was a radiopaque shadow at the level of the 7th Cervical vertebrae - 1st Thoracal which was behind of the trachea (Fig. 1A and B). The diagnosis is ingested foreign body in upper third esophagus. Because the patient is a neonate, the patient was then referred to a referral hospital in West Java for foreign body removal with endoscopy. At the time in the Emergency Department at the referral hospital, the patient still looked calm, no shortness of breath, and O_2_ saturation is 99% with normal air. The patient was prepared for foreign body extraction, and nil by mouth for 4 h by administering intravenous fluids to prevent dehydration.

**Fig. 2:**
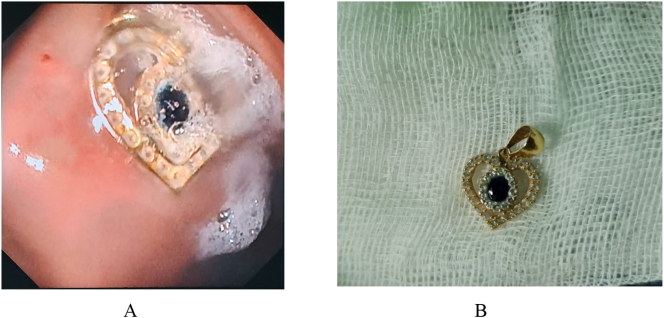
A. The foreign body in upper third esophagus, B. The pendant, diameter 1,9 cm.

The foreign body was removed 4 h later by a senior pediatric surgeon using a flexible endoscope under general anesthesia. The interval for the foreign body removal to the incident is 30 h. A heart-shaped golden and gemstone neck pendant was found in the upper third of the esophagus, measuring 1.9 cm in diameter. No signs of bleeding or inflammation were found in the esophageal lumen (Fig. 2A and B). The patient was discharged after 24-hour observation in good condition. The patient went to the pediatric surgery outpatient clinic 6 weeks later without any complaints of stridor, nor swallowing difficulties. Doctors have educated the parents to be careful in storing small items and supervise children while playing in the future.

## 3 Discussion

Foreign body ingestion is a common problem in the pediatric population occurring in children aged 6 months to 5 years up to 75% of cases [1], [5]. Foreign body in children aged under 6 months old is very rare; we only found 4 cases in neonates and 2 cases of esophageal foreign body in infant aged 2 months old with esophageal obstruction and respiratory distress [6], [7], [8], [9], [10], [11]. Our patient is the youngest one reported with ingested foreign body, and this case is unique and unusual in a 28-day-old neonate without any symptoms. Diagnosis is very difficult in neonates because the diet consists of full fluids which will easily pass the obstruction. This is consistent with our patient who does not has symptom of dysphagia [7]. A high index of suspicion for foreign body ingestion must be suspected in unattended children from lower socio-economic status and babies whose presence was culturally considered as an inauspicious birth star [6]. Foreign body ingestion is diagnosed primarily based on witness statements and from radiological examination which should cover the neck, the chest and the entire abdomen [12].

Most ingested foreign bodies would pass through the gastrointestinal system spontaneously, or they may become impacted. The most common impaction site is at the esophagus, at the level of the cricopharyngeus muscle, accounting for over 75% of all cases of foreign body impaction [5]. Foreign body impaction may result in complications such as mucosal abrasions, bleeding, gastrointestinal perforation, abscess or fistula formation. The impaction of foreign body is a strong indication for foreign body removal, and as an addition, 20% will require endoscopic intervention [5], [12]. In our case, the foreign body is impacted in upper third esophagus or at the thoracic inlet esophagus. On chest radiograph, the thoracic inlet is defined as the area between the two clavicles. This is the site of transition of skeletal muscle into the smooth muscle of the esophagus, about 75% all of foreign bodies are stuck in this site [12].

Sharp and elongated foreign bodies are responsible for 15% to 35% of perforations following foreign body ingestion. As a general rule, objects longer than 3 cm, wider than 2 cm and sharp in young children are unlikely to pass and should be removed endoscopically and immediately within 24 h of ingestion [11], [13]. Esophageal foreign body can damage the esophagus (perforations and strictures) because direct tissue damage by pressure and by chemical and electrical burns [9]. The foreign body in our case is a pendant with a sharp tip, which is at risk for causing bleeding and perforation of the esophagus. We planned to remove the foreign body immediately with flexible endoscopy. In intraoperative findings, we found a pendant measuring 1.9 cm in diameter, and no signs of bleeding and aberrations in the esophageal wall.

Several approaches can be the options in the case of foreign body removal, for example, the use of flexible or rigid endoscopy, McGill forceps, and Foley catheter. For the proximal esophagus, flexible endoscopy is the standard for foreign body removal in children, because it provides direct visualisation and the possibility of manipulation and the examination of potential complications in the structures of the region [1], [13].

The limitation of this case is that the extraction was done in >24 h. This is caused by the late recognition of the foreign body ingestion by the parents and the delay in the referral process to a referral hospital. A delay in the extraction can increase complications in the esophagus such as abrasions and bleeding; these conditions can increase the possibility of strictures in the future.

## 4 Conclusion

Although most ingested foreign bodies in children pass spontaneously, certain foreign bodies can be harmful if they are not meticulously treated. High index suspiciousness, witness statements, and radiological examination are the important points in diagnosing ingested foreign body in neonates. Clinicians are required to provide education to parents to supervise their children when playing together.

## Provenance and peer review

Not commissioned, externally peer-reviewed.

## Source of funding

No sponsorship for this case report.

## Ethical approval

The informed consent form was declared that patient data or samples will be used for educational or research purposes. Our institutional review board also does not provide an ethical approval in the form of case report.

## Consent

The patient in this case report is a child, and we have included informed consent from the parents so that this case can be reported and published for educational purposes. A copy of the written parental consent is available for review by the Editor-in-Chief of this journal on request.

## Author contribution

Dian Adi Syahputra, Vita Indriasari, and Fransiska Kusumowidagdo conceived the draft and final revision of the manuscript. Dikki Drajat Kusmayadi is a Senior Pediatric Surgeon who performs the operation, care leader and final revision of the manuscript.

## Research registration

Not applicable.

## Guarantor

Dian Adi Syahputra.

## Declaration of competing interest

The authors declare that there is no conflict of interest regarding publication of this paper.

## References

[1] Dorterler M.E., Gunendi T. (2020). Foreign body and caustic substance ingestion in childhood. Open Access Emerg. Med..

[2] Esparaz J.R., Carter S.R., Mathis M.S., Chen M.K., Russel R.T. (2020). Esophageal foreign body management in children: can it wait?. J. Laparoendosc. Adv. Surg. Tech..

[3] Gummin D.D., Mowry J.B., Spyker D.A. (2019). 2018 annual report of the American Association of Poison Control Centers’ National Poison Data System (NPDS): 36th annual report. Clin. Toxicol. (Phila.).

[4] Agha R.A., Franchi T., Sohrabi C., Mathew G., for the SCARE Group (2020). The SCARE 2020 guideline: updating consensus Surgical CAse REport (SCARE) guidelines. Int. J. Surg..

[5] Jayachandra S., Eslick G.D. (2013). A systematic review of paediatric foreign body ingestion: presentation, complications, and management. Int. J. Pediatr. Otorhinolaryngol..

[6] Janarthanan V., Moorthi K., Shaha K.K. (2019). Fatality due to button battery lodgment in the upper digestive tract of a neonate an unusual presentation. Am J Forensic Med Pathol.

[7] Tarnowska A., Roik D., Chmielik L.P., Brzewski M. (2010). An unusual oesophageal foreign body in neonate– case report. Pol. J. Radiol..

[8] Pujar V.C., Joshi S.S., Dhad S.M. (2013). Unusual cause of esophageal obstruction in a neonate presenting as esophageal atresi. J. Neonatal Surg..

[9] Singh R., Pandit C., Gupta D., Vajifdar H. (2021). Foreign body esophagus in a neonate: unusual age and unusual presentation. J. Anaesthesiol. Clin. Pharmacol..

[10] Lone S.A., Hameed A., Shiekh F.A. (2021). Foreign body esophagus in a young infant. Clin. Case Rep..

[11] Bahadur S., Bhatia R. (1983). Impacted esophageal foreign body in an infant. Indian J. Pediatr..

[12] A-Kader H.H. (2010). Foreign body ingestion: children like to put objects in their mouth. World J. Pediatr..

[13] Bekkerman M., Sachdev A.H., Andrade J., Twersky Y., Iqbal S. (2016). Endoscopic management of foreign bodies in the gastrointestinal tract: a review of the literature. Gastroenterol. Res. Pract..

